# High *O*-linked *N*-acetylglucosamine transferase expression predicts poor survival in patients with early stage lung adenocarcinoma

**DOI:** 10.18632/oncotarget.25772

**Published:** 2018-07-24

**Authors:** Yi-Cheng Lin, Chia-Hung Lin, Yi-Chen Yeh, Hsiang-Ling Ho, Yu-Chung Wu, Mei-Yu Chen, Teh-Ying Chou

**Affiliations:** ^1^ Department of Pathology and Laboratory Medicine, Taipei Veterans General Hospital, Taipei 11221, Taiwan; ^2^ Institute of Biochemistry and Molecular Biology, National Yang-Ming University, Taipei 11221, Taiwan; ^3^ School of Medicine, National Yang-Ming University, Taipei 11221, Taiwan; ^4^ Division of Thoracic Surgery, Department of Surgery, Taipei Veterans General Hospital, Taipei 11221, Taiwan; ^5^ Genome Research Center, National Yang-Ming University, Taipei 11221, Taiwan; ^6^ Institute of Clinical Medicine, National Yang-Ming University, Taipei 11221, Taiwan

**Keywords:** lung cancer, OGT, O-GlcNAcylation, EGFR, prognostic marker

## Abstract

Tumor cell heterogeneity can make selection of appropriate interventions to lung cancer a challenge. Novel biomarkers predictive of disease risk and treatment response are needed to improve personalized treatment strategies. *O*-GlcNAcylation, the attachment of *β*-*N*-acetylglucosamine (*O*-GlcNAc) to serine or threonine residues of intracellular proteins, modulates protein functions and is implicated in cancer pathogenesis. *O*-GlcNAc-transferase (OGT) and *O*-GlcNAcase (OGA) catalyze *O*-GlcNAc addition and removal, respectively. We used immunohistochemistry to explore the utility of OGT, OGA, and *O*-GlcNAc as potential biomarkers for lung adenocarcinoma. We found that high OGT expression is associated with poor overall survival (OS) in both stage I patients (*P*=0.032) and those at variable stages of disease (*P*=0.029), and with poor recurrence-free survival (RFS) in stage I patients (*P*=0.035). High OGT expression is also associated with poorer OS in patients with *EGFR* wild-type tumors at variable stages (*P*=0.038). Multivariate analysis indicated that OGT expression is an independent prognostic factor for RFS (HR 2.946, 95% CI: 1.411–6.150, *P*=0.004) and OS (HR 2.002, 95% CI: 1.183–3.391, *P*=0.010) in stage I patients. Our findings indicate OGT is a promising biomarker for further classifying early stage lung adenocarcinomas.

## INTRODUCTION

Cancer cells reprogram their metabolism in order to promote cell growth, survival, and proliferation, which is known as the Warburg effect. The common characteristic of this effect is a shift of oxidative phosphorylation to aerobic glycolysis for energy production, which drives the increase of glucose uptake and hexosamine biosynthesis pathway (HBP) flux [1]. This cancer-specific metabolism was found to associate with elevated *O*-GlcNAcylation in various human malignancies including breast, prostate, and colorectal cancers.

*O*-GlcNAcylation is an inducible and dynamic post-translational modification in which N-acetylglucosamine (GlcNAc) is attached to serine or threonine residues in nuclear and cytoplasmic proteins [2, 3] Similar to phosphorylation, *O*-GlcNAcylation modulates protein functions, thereby regulating a myriad of fundamental cellular processes, including gene expression, cellular signaling, and metabolism [2, 4]. In human cells, a pair of *O*-GlcNAc cycling enzymes maintains cellular *O*-GlcNAcylation homeostasis: *O*-GlcNAc transferase (OGT) transfers the GlcNAc moiety from the donor substrate, uridine diphosphate GlcNAc (UDP-GlcNAc), onto target protein serine or threonine residues, while *O*-GlcNAcase (OGA) removes the modification [5]. Disruption in *O*-GlcNAc homeostasis may promote the development of human cancers through modification of several key players in tumorigenesis and cancer progression, such as p53, c-Myc, Snail, etc. [6–8].

Lung cancer is the most common cause of cancer death worldwide [9], with non-small cell lung cancers (NSCLCs) accounting for 85–90% of all cases [10]. Among different histological subtypes of NSCLC, adenocarcinoma is the most common primary lung malignancy [10, 11]. Although the discovery of oncogenic driver mutations and their subsequent association to specific targeted therapies ushered in an era of personalized medicine for lung cancer patients, there remains a pressing need for novel biomarkers. The role of *O*-GlcNAcylation remains largely unexplored in lung cancer, and only a few studies have provided evidence for its significance.

OGT silencing in lung cancer cells reduced colony formation in soft agar colony assays and inhibited invasion in transwell assays [12]. An immunohistochemistry (IHC) analysis of lung squamous cell carcinoma tissues showed elevated *O*-GlcNAcylation and OGT expression in cancer tissues compared with adjacent non-cancerous tissues [12]. Glucose-6-phosphate dehydrogenase (G6PD), the rate-limiting enzyme of the pentose phosphate pathway, was found to be modified and activated by *O*-GlcNAcylation in response to hypoxia, and the level of *O*-GlcNAcylated G6PD was higher in lung cancers than in matching normal lung tissue [13]. In addition, recent analyses of *OGT* and *OGA* expression using the Oncomine cancer microarray database found elevated *OGT* mRNA in lung adenocarcinoma tissues compared with normal lung tissues in most datasets [14].

Taken together, evidence from previous studies point out that *O*-GlcNAcylation may participate in carcinogenesis of lung cancer. However, there are as yet no reports regarding clinical impacts of *O*-GlcNAcylation on lung cancer patients. In this study, the expression of OGT, OGA, and *O*-GlcNAc were examined in lung adenocarcinoma tissues using immunohistochemistry, and the clinicopathological features as well as patients’ outcome were evaluated to assess the prognostic relevance of these markers.

## RESULTS

### General patient characteristics

**Figure 1:**
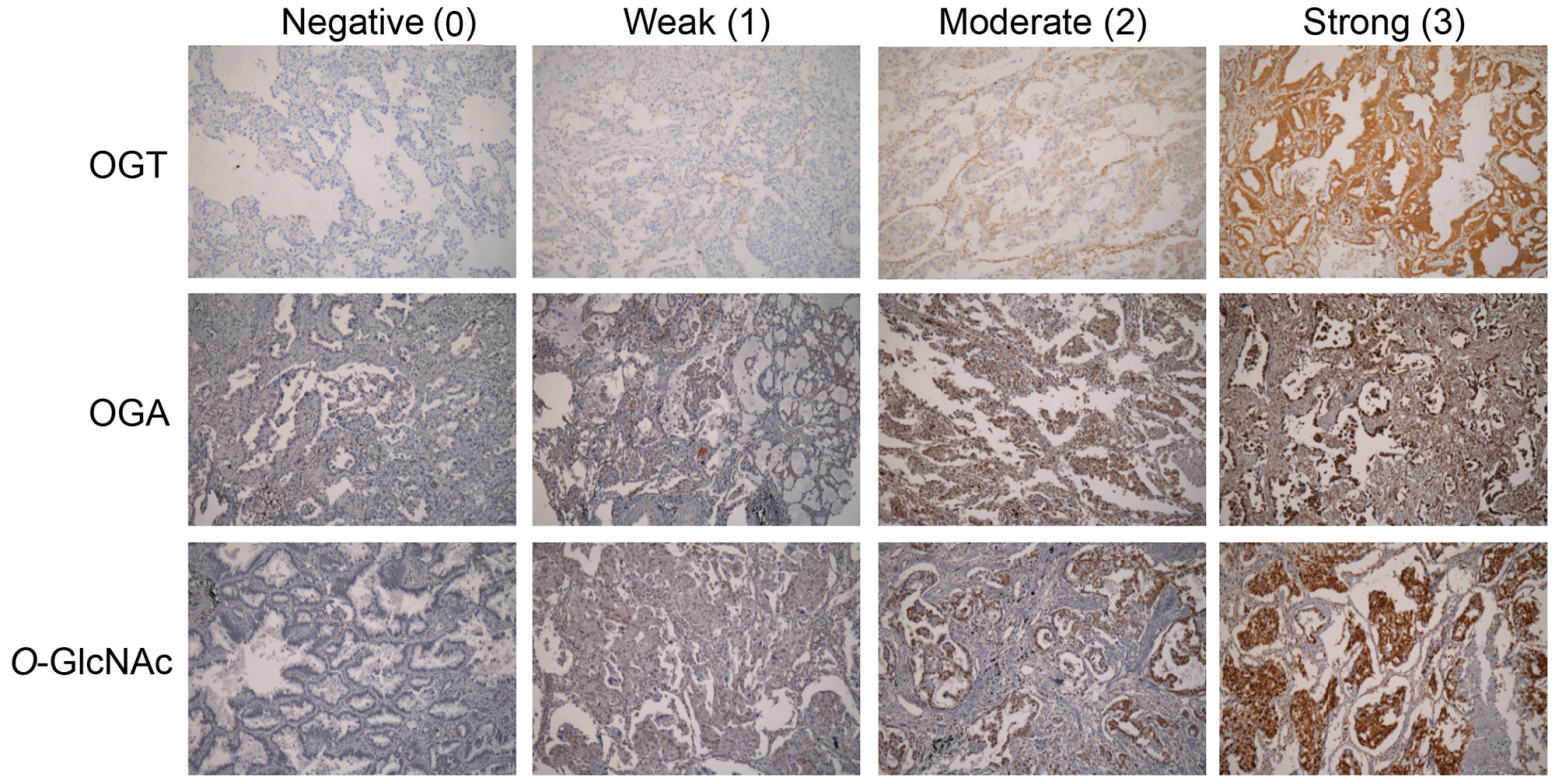
OGT and OGA expression and scoring, and *O*-GlcNAc levels in lung cancer samples Representative IHC images from each TMA. Scores indicated weak, moderate, and strong positive staining in tumors.

The cohort A tissue microarray (TMA) included tumor tissues from 117 patients with stage I lung adenocarcinoma. The median age of cohort A patients was 69 years (range, 35–87 years; mean, 66.5 years). Follow-up was available in all cases and ranged from 0.23 to 126 months (median, 65.37 months; mean, 58.8 months). During the follow-up period, 39 (33.3%) patients presented with evidence of disease recurrence. The total survival rate was 57.6% at 5 years and 35.6% at 10 years. *EGFR* mutation status was available for 108 tumors, 68 (63.0%) of which had activating mutations. The expressions of OGT, OGA and *O*-GlcNAc in both cohorts were examined using immunohistochemistry, and the representative staining images were shown in Figure 1. The cut-off values for determining high and low expression of IHC stains were selected using time-dependent receiver operating characteristic (ROC) curves analysis. We examined the correlation between protein levels and clinicopathological features. Neither OGA nor *O*-GlcNAc staining correlated with age, sex, recurrence, or tumor size (Table 1). However, OGT expression was higher in non-smokers than smokers (*P*=0.008), and tumors in the high-OGT subgroup displayed better differentiation than those in the low-OGT subgroup (*P*=0.018). *EGFR* mutations were associated with neither OGT, OGA nor *O*-GlcNAc expression.

**Table 1:** The association of OGT, OGA or *O*-GlcNAc levels and clinicopathological features of patients in cohort A

Clinicopathological characteristics (Total *n* = 117)	OGT		*P* values	OGA		*P* values	*O*-GlcNAc		*P* values
	Low (*n* = 54)	High (*n* = 63)		Low (*n* = 68)	High (*n* = 49)		Low (*n* = 47)	High (*n* = 70)	
Age (years)									
Mean (±SD)	67.6 (±10.6)	65.6 (±9.0)	0.279^a^	66.5 (±8.5)	66.6 (±11.4)	0.984^a^	65.5 (±11.1)	67.2 (±8.8)	0.358^a^
Range	35-87	40-80		42-87	35-87		40-87	35-82	
Sex									
Male	40 (74.1%)	37 (58.7%)	0.081^b^	46 (67.7%)	31 (63.3%)	0.622^b^	34 (72.3%)	43 (61.4%)	0.223^b^
Female	14 (25.9%)	26 (41.3%)		22 (32.3%)	18 (36.7%)		13 (27.7%)	27 (38.6%)	
Smoking status^†^									
Non-smoker	19 (35.9%)	36 (61.0%)	0.008^b**^	33 (49.3%)	22 (48.9%)	0.970^b^	26 (57.8%)	29 (43.3%)	0.133^b^
Smoker	34 (64.1%)	23 (39.0%)		34 (50.7%)	23 (51.1%)		19 (42.2%)	38 (56.7%)	
Unknown	1	4		1	4		2	3	
Recurrence status^†^									
No	39 (75.0%)	36 (58.1%)	0.058^b^	47 (72.3%)	28 (57.1%)	0.091^b^	33 (70.2%)	42 (62.7%)	0.404^b^
Yes	13 (25.0%)	26 (41.9%)		18 (27.7%)	21 (42.9%)		14 (29.8%)	25 (37.3%)	
Unknown	2	1		3	0		0	3	
Tumor size (cm)									
Mean (±SD)	3.01 (±1.12)	3.33 (±1.13)	0.133^a^	3.18 (±1.22)	3.19 (±1.00)	0.982^a^	3.08 (±1.12)	3.25 (±1.14)	0.436^a^
Range	1.0-5.5	1.5-6.0		1.5-6.0	1.0-5.5		1.0-6.0	1.5-6.0	
Tumor differentiation									
Well to moderate	29 (53.7%)	47 (74.6%)	0.018^b*^	45 (66.2%)	31 (63.3%)	0.745^b^	33 (70.2%)	43 (61.4%)	0.329^b^
Poor	25 (46.3%)	16 (25.4%)		23 (33.8%)	18 (36.7%)		14 (29.8%)	27 (38.6%)	
Tumor necrosis									
No	31 (57.4%)	37 (58.7%)	0.885^b^	42 (61.8%)	26 (53.1%)	0.346^b^	31 (66.0%)	37 (52.9%)	0.159^b^
Yes	23 (42.6%)	26 (41.3%)		26 (38.2%)	23 (46.9%)		16 (34.0%)	33 (47.1%)	
Angiolymphatic invasion									
No	35 (64.8%)	34 (54.0%)	0.234^b^	41 (60.3%)	28 (57.1%)	0.732^b^	28 (59.6%)	41 (58.6%)	0.914^b^
Yes	19 (35.2%)	29 (46.0%)		27 (39.7%)	21 (42.9%)		19 (40.4%)	29 (41.4%)	
EGFR status									
Wild-type	23 (46.0%)	17 (29.3%)	0.073^b^	25 (40.3%)	15 (32.6%)	0.412^b^	13 (31.0%)	27 (40.9%)	0.296^b^
Mutant	27 (54.0%)	41 (70.7%)		37 (59.7%)	31 (67.4%)		29 (69.0%)	39 (59.1%)	
Unknown	4	5		6	3		5	4	
Histiological subtype^†^									
Lepidic	4	5	0.028^b*^	7	2	0.233^b^	6	3	0.546^b^
Acinar	14	29		24	19		17	26	
Papillary	8	7		11	4		5	10	
Micropapillary	9	13		9	13		8	14	
Solid	17	6		13	10		9	14	
Unknown	2	3		4	1		2	3	
Histiological subtype group^†^									
Lepidic/acinar/papillary	26 (50.0%)	41 (68.3%)	0.048^b*^	42 (65.6%)	25 (52.1%)	0.148^b^	28 (62.2%)	39 (58.2%)	0.671^b^
Micropapillary/solid	26 (50.0%)	19 (31.7%)		22 (34.4%)	23 (47.9%)		17 (37.8%)	28 (41.8%)	
Unknown	2	3		4	1		2	3	

Abbreviation: SD, standard deviation. ^a^. independent samples *t*-test ^b^. two-tailed Pearson’s chi-squared test ^†^. Patients with unknown status were excluded in the analysis ^*^, *P* < 0.05; ^**^, *P* <0.01.

The cohort B TMA included tumor tissues from 201 patients with lung adenocarcinoma at various stages. Cohort B patient median age was 67 years and the median follow-up was 53.67 months. Stage I lung cancer patients accounted for 56.2% (113/201) of this cohort. *EGFR* mutation status was available for 172 tumors, 96 (55.8%) of which had activating mutations. We found no associations between OGT, OGA, or *O*-GlcNAc staining and patient age, sex, tumor stage, or *EGFR* mutation status (Table 2).

**Table 2:** The association of OGT, OGA or *O*-GlcNAc levels and clinicopathological features of patients in cohort B

Clinicopathological characteristics (Total *n* =201)	OGT		*P* values	OGA		*P* values	*O*-GlcNAc		*P* values
	Low (n = 94)	High (*n* = 107)		Low (*n* = 148)	High (n = 53)		Low (*n* = 51)	High (*n* = 150)	
Age (years)									
Mean (±SD)	65.0 (±11.9)	65.7 (±10.4)	0.661^a^	65.0 (±11.2)	66.6 (±10.9)	0.361^a^	63.8 (±11.7)	65.9 (±10.9)	0.238^a^
Range	35-88	38-83		35-83	37-88		37-83	35-88	
Sex									
Male	52 (55.3%)	64 (59.8%)	0.520^b^	83 (56.1%)	33 (62.3%)	0.434^b^	29 (56.9%)	87 (58.0%)	0.887^b^
Female	42 (44.7%)	43 (40.2%)		65 (43.9%)	20 (37.7%)		22 (43.1%)	63 (42.0%)	
Follow-up time, months									
Mean (±SD)	54.1 (±22.5)	47.4 (±26.1)	0.056^a^	52.8 (±24.0)	44.2 (±25.6)	0.029^a*^	54.8 (±29.1)	49.1 (±22.9)	0.158^a^
Range	3.03-99.97	1.20-100.43		4.63-100.43	1.20-89.50		3.53-99.97	1.20-100.43	
Disease stage^†^									
Stage I	59	54	0.181^b^	86	27	0.823^b^	29	84	0.482^b^
Stage II	6	12		12	5		7	11	
Stage III	22	35		40	17		12	45	
Stage IV	6	4		8	2		2	8	
Unknown	1	2		1	2		1	2	
Disease stage group^†^									
Stage I	59 (63.4%)	54 (51.4%)	0.088^b^	86 (58.5%)	27 (52.9%)	0.489^b^	29 (58.0%)	84 (56.8%)	0.878^b^
Stage II/III/IV	34 (36.6%)	51 (48.6%)		61 (41.5%)	24 (47.1%)		21 (42.0%)	64 (43.2%)	
Unknown	1	2		1	2		1	2	
EGFR status^†^									
Wild-type	32 (40.5%)	44 (47.3%)	0.370^b^	59 (46.1%)	17 (38.6%)	0.390^b^	23 (54.8%)	53 (40.8%)	0.112^b^
Mutant	47 (59.5%)	49 (52.7%)		69 (53.9%)	27 (61.4%)		19 (45.2%)	77 (59.2%)	
Unknown	15	14		20	9		9	20	
Histiological subtype^†^									
Lepidic	18	14	0.287^b^	23	9	0.056^b^	9	23	0.197^b^
Acinar	31	45		59	17		24	52	
Papillary	19	13		27	5		10	22	
Micropapillary	11	11		11	11		3	19	
Solid	14	21		24	11		5	30	
Unknown	1	3		4	0		0	4	
Histiological subtype group^†^									
Lepidic/acinar/papillary	68 (73.1%)	72 (69.2%)	0.548^b^	109 (75.7%)	31 (58.5%)	0.018^b*^	43 (84.3%)	97 (66.4%)	0.015^b*^
Micropapillary/solid	25 (26.9%)	32 (30.8%)		35 (24.3%)	22 (41.5%)		8 (15.7%)	49 (33.6%)	
Unknown	1	3		4	0		0	4	

SD, standard deviation. ^a^. independent samaples *t*-test ^b^. two-tailed Pearson’s chi-squared test ^†^, Patients with unknown status were excluded in the analysis ^*^, *P* <0.05.

### Association between *O*-GlcNAc level and lung adenocarcinoma histological subtypes

The 2011 IASLC/ATS/ERS lung adenocarcinoma classification guidelines categorize tumors into subtypes with prognostic differences according to their predominant histological patterns [15, 16]. The low-grade adenocarcinoma *in situ* (AIS) and minimally invasive adenocarcinoma (MIA) subtypes are associated with very low risk of disease recurrence if tumors are completely resected. The invasive adenocarcinomas include low to intermediate-grade (lepidic, acinar, and papillary) subtypes and high-grade (micropapillary and solid) subtypes, which are respectively associated with a relatively low and high risk of recurrence and cancer-related death.

We investigated associations between *O*-GlcNAc or cycling enzymes in tumors and histological subtypes. The expression level of OGT was statistically different between different histological subtypes of lung adenocarcinoma (*P*=0.028, Table 1). When histological subtypes were grouped into low to intermediate- and high-grade subgroups for comparison, we observed high OGT levels in lepidic/acinar/papillary histological subtype tumors in cohort A (*P*=0.048; Table 1). In cohort B, high OGA or *O*-GlcNAc levels were observed more frequently in micropapillary/solid histological subtype tumors (*P*=0.018 and 0.015, respectively; Table 2).

### Association between high OGT expression and poorer patient survival

**Figure 2:**
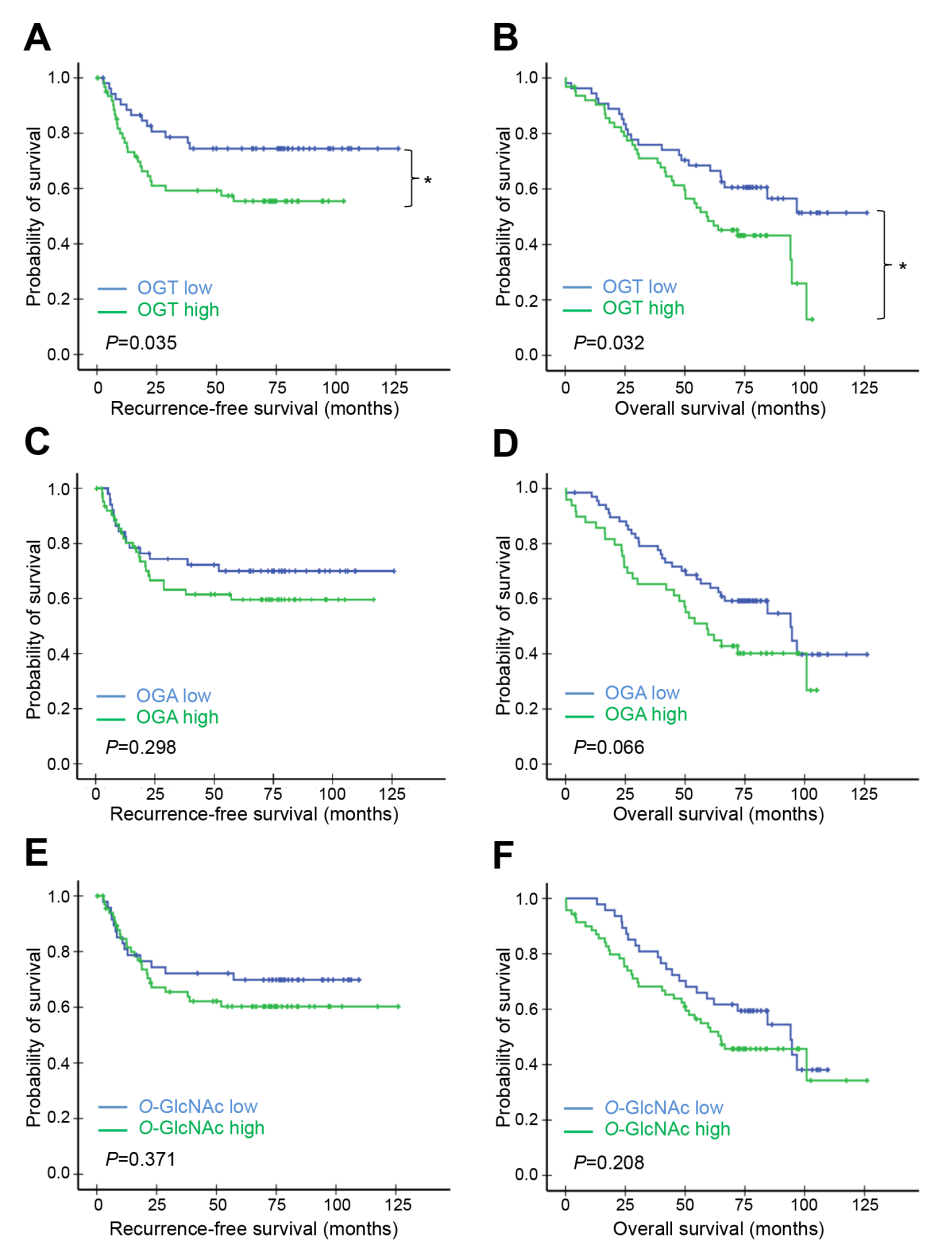
Kaplan–Meier survival analysis of patients in cohort A according to OGT, OGA, or *O*-GlcNAc levels Patients in cohort A (*n*=117) were separated into high and low OGT, OGA, or *O*-GlcNAc expression groups using IHC score cut-off values (estimated from time-dependent ROC curves at *t*=120 months). RFS **(A, C)** & **(E)** and OS **(B, D)**, & **(F)** curves were plotted for each group. *P*-values derived from the log-rank test were indicated in each comparison. ^*^P<0.05; ^**^P<0.01.

**Figure 3:**
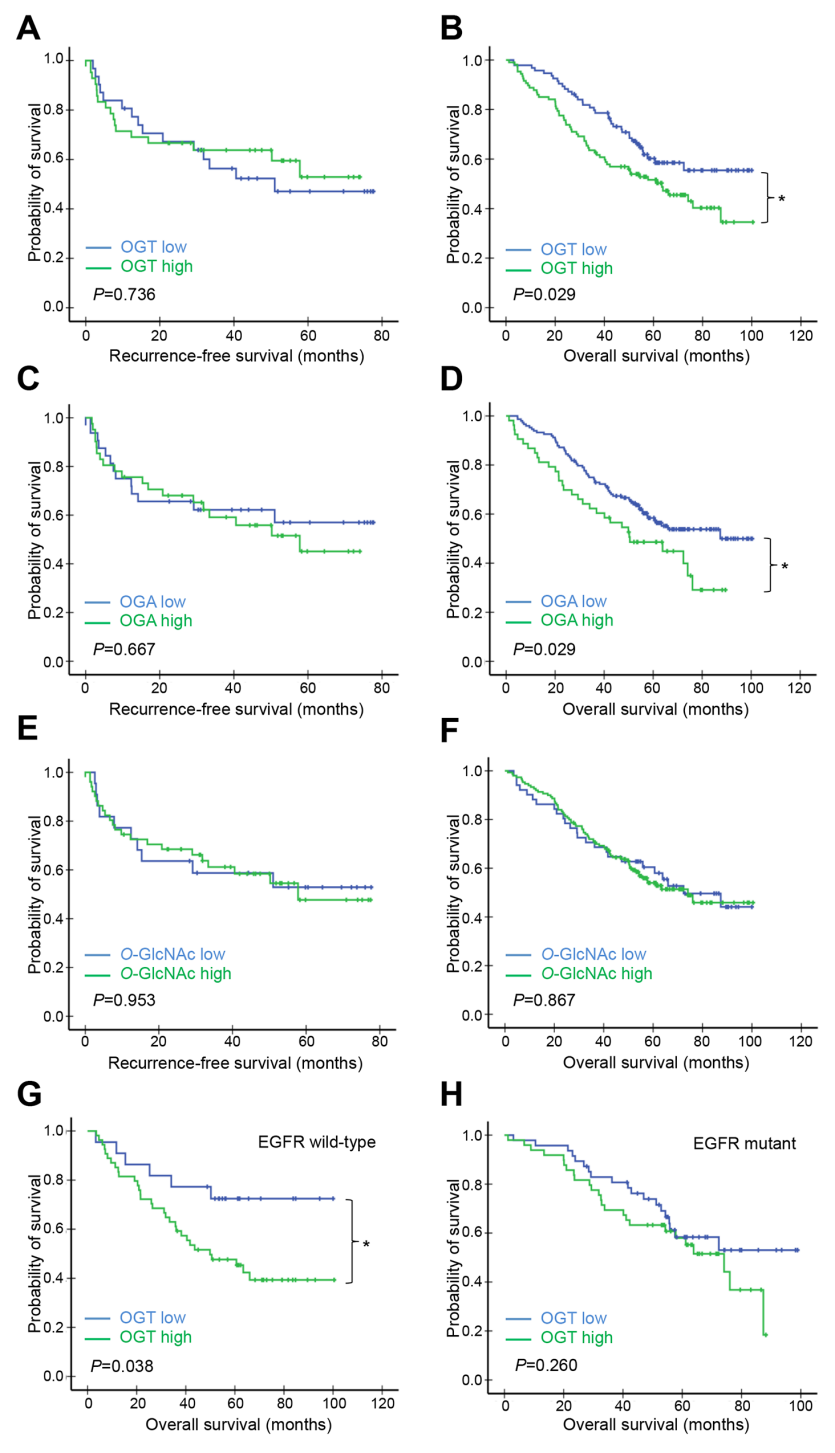
Kaplan–Meier survival analysis of patients in cohort B according to OGT, OGA, or *O*-GlcNAc levels Patients in cohort B (*n*=201) were separated into high and low OGT, OGA, or *O*-GlcNAc expression groups using IHC score cut-off values (estimated from time-dependent ROC curves at *t*=120 months) **(A–F)** Patients were first divided into *EGFR* wild-type **(G)** and mutant **(H)** groups. Each group was further divided into high and low OGT subgroups using IHC score cut-off values (estimated from time-dependent ROC curves at *t*=120 months). RFS (A, C, E, & G) and OS (B, D, F, & H) curves were plotted for each group. *P*-values derived from the log-rank test were indicated in each comparison. ^*^P<0.05.

The associations between OGT, OGA, or *O*-GlcNAc levels and patient survival were also investigated using the Kaplan–Meier method and the log-rank test. The high-OGT subgroup in cohort A had shorter recurrence-free survival (RFS) (*P*=0.035; Figure 2A) and overall survival (OS) (*P*=0.032; Figure 2B) in comparison with the low-OGT subgroup. However, the RFS and OS did not show significant difference between the low- and high-OGA subgroups (Figure 2C–2D), or the low- and high *O*-GlcNAc subgroups (Figure 2E–2F). In cohort B, the high-OGT and high-OGA subgroups had shorter OS times compared with the low-OGT (*P*=0.029; Figure 3B) and low-OGA subgroups (*P*=0.029; Figure 3D), respectively. No significant differences were noted in any other comparisons (Figure 3A, 3C, 3E, and 3F).

Since *EGFR* mutation is an important oncogenic driver mutations in NSCLC, we further investigated whether or not *EGFR* mutation status associates with the prognostic performance of OGT, OGA, or *O*-GlcNAc [17]. In cohort A, high expression of OGT, OGA or *O*-GlcNAc did not significantly associate with OS when patients were further grouped into *EGFR* mutant and wild-type (Supplementary Figure 1A-1F). In cohort B, high OGT expression in tumors was associated with shorter OS in the *EGFR* wild-type group (*P*=0.038; Figure 3G), but not the *EGFR* mutant group (Figure 3H). Neither OGA nor *O*-GlcNAc levels were associated with OS in either *EGFR* subgroup (Supplementary Figure 2A–2D).

### Correlation between OGT and OGA expression in lung adenocarcinomas

*O*-GlcNAcylation homeostasis requires tight and coordinated regulation of OGT and OGA; inhibiting OGT downregulates OGA and vice versa [18, 19]. We assessed relationships between OGT, OGA, and *O*-GlcNAc levels in lung adenocarcinoma tissues using the Spearman rank correlation analysis. OGT and OGA levels were positively correlated in both cohort A (*r*=0.430, *P*<0.001) and B (*r*=0.192, *P*=0.006). OGT and *O*-GlcNAc (*r*=0.264, *P*=0.004), and OGA and *O*-GlcNAc levels (*r*=0.245, *P*=0.008) were only positively associated in cohort A, but not cohort B (*r*=0.053, *P*=0.451 for OGT and *O*-GlcNAc; *r*=0.138, *P*=0.051 for OGA and *O*-GlcNAc). We also analyzed relationships between these three markers separately in cohort B *EGFR* wild-type and mutant groups. While no correlations were observed between any two of the three markers in the *EGFR* wild-type group, OGT and OGA (*r*=0.207,*P*=0.043), and OGA and *O*-GlcNAc levels (*r*=0.228, *P*=0.026) were positively correlated in the *EGFR* mutant group.

**Figure 4:**
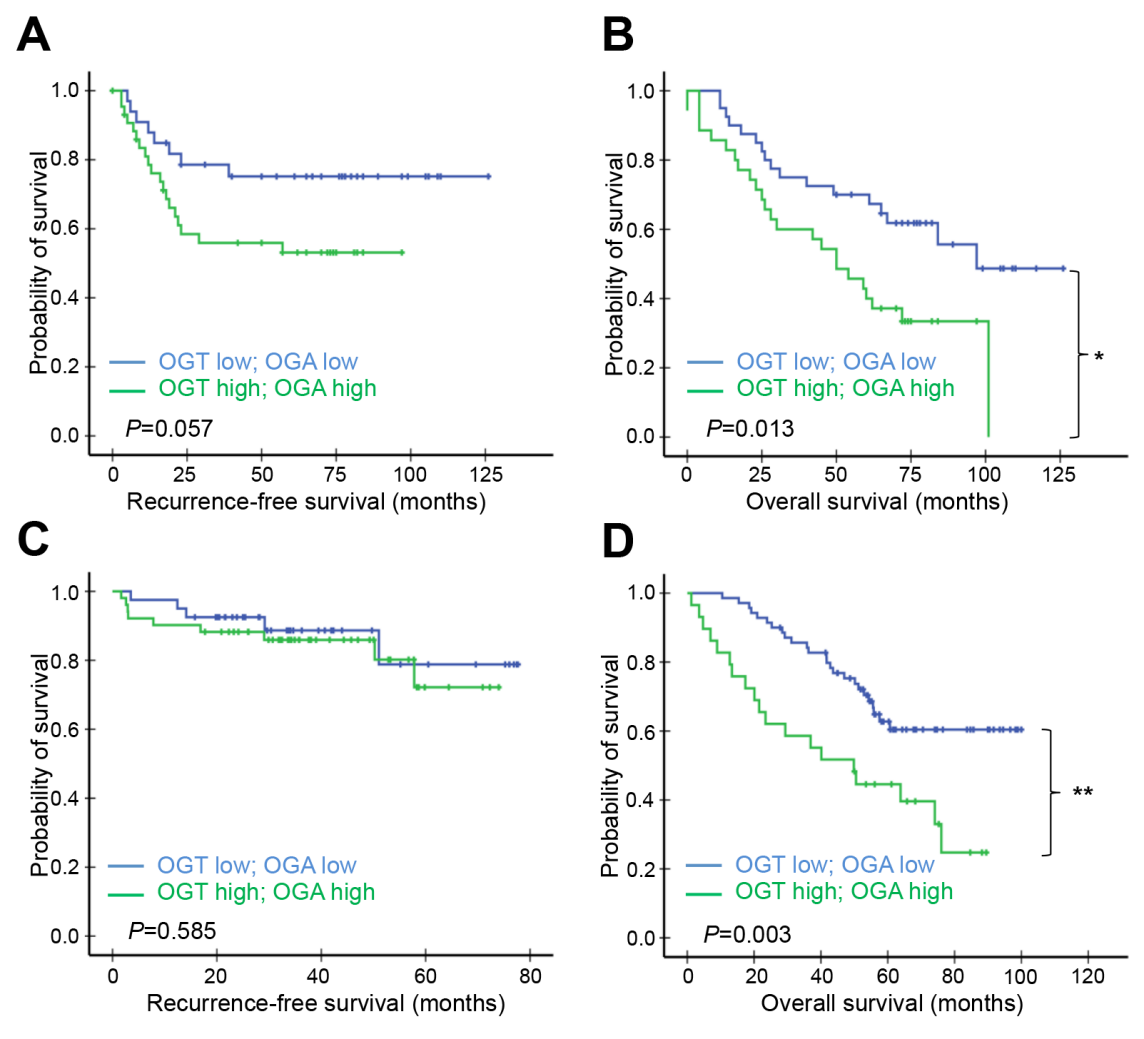
Kaplan–Meier survival analysis of patients according to both OGT and OGA levels Patients in cohort A **(A, B)** or B **(C, D)** whose lung adenocarcinomas expressed both OGT and OGA at high or low levels were compared. RFS (A & C) and OS (B & D) curves were plotted for each group. *P*-values derived from the log-rank test were indicated in each comparison. ^*^P<0.05.

Given the positive correlation between OGT and OGA expression in the two TMAs, we further compared survival in high-OGT/high-OGA (high/high) and low-OGT/low-OGA (low/low) subgroups from both cohorts. The cohort A high/high subgroup had shorter RFS (*P*=0.057; Figure 4A) and OS times (*P*=0.013; Figure 4B) than the low/low subgroup. The cohort B high/high and low/low subgroups had similar RFS times (*P*=0.585; Figure 4C), but the high/high subgroup had a shorter OS time than the low/low subgroup (*P*=0.003; Figure 4D). OGT and OGA levels were positively correlated in the cohort B *EGFR* mutant group, but OS was the same in high/high and low/low subgroup patients (Supplementary Figure 2E).

### OGT expression is an independent prognostic factor in patients with early stage lung adenocarcinoma

We performed a Cox proportional hazards regression analysis to identify prognostic factors for RFS and OS in lung adenocarcinoma patients. Univariate analysis showed that tumor necrosis, tumor differentiation, histological subtype, and OGT expression influenced RFS in cohort A patients (Table 3). Multivariate regression revealed that OGT expression was the only independent prognostic factor for RFS (hazard ratio (HR) 2.946, 95% confidence interval (CI): 1.411–6.150, *P*=0.004; Table 3). Univariate analysis showed that age, tumor necrosis, and OGT expression were associated with reduced OS in cohort A patients. Multivariate analysis showed that all three factors were independently predictive of OS, with a hazard ratio of 2.037 for age (95% CI: 1.137–3.651, *P*=0.017), 1.840 for tumor necrosis (95% CI: 1.104–3.068, *P*=0.019), and 2.002 for OGT expression (95% CI: 1.183–3.391, *P*=0.010) (Table 3). Univariate analysis identified disease stage as the only factor associated with poor RFS in cohort B (HR 6.125, 95% CI: 2.936–12.782, *P*=0.000), while gender, disease stage, histological subtype, OGA, and OGT were associated with poor OS; however, multivariate analysis excluded both OGA and OGT levels as independent prognostic factors for OS (Supplementary Table 1). Together, our data suggest that OGT expression may serve as an independent prognostic factor for RFS and OS in patients with early stage lung adenocarcinoma.

**Table 3:** Univariate and multivariate Cox regression analysis of recurrence-free survival and overall survival of patients in cohort A

Characteristics	Comparison	Univariate analysis		Multivariate analysis	
		HR (95%CI)	*P* values	HR (95%CI)	*P* values
**Recurrence-Free Survival**					
Age	≦65 years; >65 years	1.656 (0.824-3.329)	0.156		
Gender	Male; Female	1.090 (0.567-2.098)	0.796		
Smoking	No; Yes	0.963 (0.501-1.850)	0.909		
Tumor size	≦3 cm; >3 cm	1.366 (0.725-2.575)	0.335		
Tumor necrosis	No; Yes	2.818 (1.486-5.343)	0.002^**^	2.055 (0.963-4.387)	0.063
Angiolymphatic invasion	No; Yes	1.414 (0.754-2.650)	0.280		
Tumor differentiation	Well and moderate; Poor	2.022 (1.076-3.797)	0.029^*^	1.613 (0.738-3.523)	0.231
Histological subtype group	Lepidic/acinar/papillary; Micropapillary/solid	2.130 (1.115-4.069)	0.022^*^	1.637 (0.782-3.427)	0.191
OGT	Low; High	2.019 (1.037-3.932)	0.039^*^	2.946 (1.411-6.150)	0.004^**^
OGA	Low; High	1.406 (0.737-2.680)	0.301		
*O*-GlcNAc	Low; High	1.347 (0.700-2.592)	0.373		
**Overall Survival**					
Age	≦65 years; >65 years	2.082 (1.174-3.691)	0.012^*^	2.037 (1.137-3.651)	0.017^*^
Gender	Male; Female	0.969 (0.567-1.657)	0.909		
Tumor size	≦3 cm; >3 cm	1.299 (0.781-2.159)	0.313		
Smoking	No; Yes	1.213 (0.722-2.039)	0.466		
Tumor necrosis	No; Yes	1.979 (1.196-3.277)	0.008^**^	1.840 (1.104-3.068)	0.019^*^
Angiolymphatic invasion	No; Yes	1.272 (0.766-2.113)	0.353		
Tumor differentiation	Well and moderate; Poor	1.525 (0.912-2.552)	0.108		
Histological subtype group	Lepidic/acinar/papillary; Micropapillary/solid	1.597 (0.949-2.688)	0.078		
OGT	Low; High	1.765 (1.044-2.985)	0.034^*^	2.002 (1.183-3.391)	0.010^*^
OGA	Low; High	1.596 (0.966-2.639)	0.068		
*O*-GlcNAc	Low; High	1.395 (0.829-2.347)	0.210		

Abbreviations: HR, hazard ratio; 95% CI, 95% confidence interval. ^*^, *P* < 0.05; ^**^, *P* < 0.01.

## DISCUSSION

In this study, we examined 318 lung adenocarcinomas including two independent TMAs, one comprising of stage I cancers and the other tumors at various clinical stages, to evaluate associations between OGT, OGA, and cellular *O*-GlcNAcylation and both clinicopathological parameters and patient outcome. Our data indicate that high expression of OGT independently predicts poor survival outcomes of patients with stage I lung adenocarcinomas. Elevated *O*-GlcNAcylation and/or altered expression of its cycling enzymes were/was previously observed in nearly all cancer types, but few studies have demonstrated the prognostic values of these *O*-GlcNAcylation markers. OGT overexpression was associated with prostate cancer progression and recurrence, and high *O*-GlcNAc IHC staining was an independent prognostic factor for poor survival [20, 21]. Increased *O*-GlcNAcylation was also associated with poor survival in cholangiocarcinoma patients [22], while *OGA* downregulation predicted recurrence in hepatocellular carcinoma after liver transplantation [23]. Our retrospective study suggested that OGT may be a promising prognostic biomarker in early stage lung adenocarcinomas. Further validation studies using larger prospective cohorts and clinical trials are required to confirm our findings.

We also observed a positive correlation between OGT and OGA levels in lung adenocarcinoma, and high levels of both enzymes predicted poor patient outcomes. Considering that hyper-*O*-GlcNAcylation is a general feature of cancer, a positive correlation between two enzymes responsible for opposite *O*-GlcNAcylation functions may seem counter-intuitive. One of the possible explanations is that the high level of OGA expression may result from a feedback mechanism of elevated OGT expression in order to maintain the homeostasis of *O*-GlcNAcylation, and cells with high levels of both OGT and OGA would likely undergo very active *O*-GlcNAc cycling, which could indicate active proliferation, metabolism, and signaling events. We speculate highly proliferative tumors with high OGT/OGA levels may be predictive of rapid disease progression and dismal patient outcomes. Consistently, our findings that only OGT, but not OGA or *O*-GlcNAc level in tumors independently predicts survival implies that the key lever in tipping *O*-GlcNAcylation homeostasis of lung adenocarcinoma is OGT expression. However, the mechanism responsible for OGT upregulation in lung adenocarcinoma remains to be determined.

In NSCLC, *EGFR* mutation is one of the driver mutations essential for tumorigenesis. Patients with *EGFR* mutations benefit most from EGFR tyrosine kinase inhibitors compared to standard chemotherapy [24]. Through analysis of *EGFR* mutation status in our cohorts, the results showed that only in cohort B but not cohort A, which composed of purely stage I patients, high OGT expression was significantly associated with poorer OS in *EGFR* wild-type patients, suggesting that their association was stage-dependent. Indeed, by analysis of OGT expression among different stages in cohort B, we found that OGT expression was higher in stage II/III/IV than in stage I (Supplementary Figure 3).

In conclusion, this study demonstrated for the first time that OGT protein expression independently predicts poor outcome in patients with early stage lung adenocarcinoma. Our findings also offer new perspectives on the role of *O*-GlcNAcylation in NSCLC. However, larger prospective cohorts are needed for validating OGT as a prognostic biomarker, and further experimental studies are required for better understanding the molecular mechanisms and clinical significance of *O*-GlcNAc cycling in NSCLC. Our work identifies OGT as a novel prognostic biomarker for classifying early stage NSCLC according to recurrence risk and to guide treatment strategy. Future investigations will determine whether or not targeting OGT is a valid therapeutic strategy for managing NSCLC.

## MATERIALS AND METHODS

### Patients and clinical data

This study was approved by the Ethics Committee of the Taipei Veterans General Hospital (Taipei-VGH), Taiwan (2014-07-001ACF). We analyzed a total of 318 archived tissue samples from two lung adenocarcinoma patient cohorts. Cohort A contained 117 patients with documented stage I lung adenocarcinoma; these patients underwent tumor resection at Taipei-VGH between 1995 and 2007. Cohort B contained 201 patients with lung adenocarcinoma at various stages, including 113 stage I, 18 stage II, 57 stage III, and 10 stage IV patients; these patients underwent tumor resection at Taipei-VGH between 2002 and 2006. Disease stage was determined according to the Union for International Cancer Control/American Joint Committee on Cancer TNM classification (7th ed.).

Data on patient demographics, clinicopathologic characteristics, and outcomes were collected retrospectively from medical records. *EGFR* mutation status was previously determined for lung adenocarcinomas in cohort B [25]. Overall survival (OS) was defined as the interval between the date of surgical resection and that of either death or last follow-up. Recurrence-free survival (RFS) was defined as the time between diagnosis and date of recurrence or death. Patients who died from other causes or for whom the cause of death was not known were censored.

### Tissue microarray

All specimens were fixed in formalin and embedded in paraffin before being archived. Hematoxylin and eosin-stained sections were evaluated microscopically by pathologists (T.-Y.C. and Y.-C.Y). 2011 International Association for the Study of Lung Cancer, American Thoracic Society, and European Respiratory Society (IASLC/ATS/ERS) classification criteria for lung adenocarcinoma were used for histologic classification [15]. Each tumor was reviewed using comprehensive histologic subtyping, and percentages of each histologic component (lepidic, acinar, papillary, micropapillary, and solid) were recorded in 5% increments. The predominant pattern was defined according to the histologic component with the greatest percentage. Other pathological parameters, including tumor differentiation, necrosis, and angiolymphatic invasion, were also evaluated in cohort A. For TMA construction, representative tumor tissue areas were selected and a 3-mm tissue core was retrieved from the paraffin block for each case.

### Immunohistochemistry analysis

Paraffin-embedded 5-μm TMA sections were deparaffinized in xylene and rehydrated via soaking in decreasing percentages of ethanol solutions (100% twice, 90% and 70% once). Sections were heated in 0.1 M citrate for antigen retrieval, treated with 3% H_2_O_2_ to block endogenous peroxidase activity, and incubated overnight at 4°C with anti-OGT (1:50; ProteinTech, Chicago, IL), anti-MGEA5 (1:100; ProteinTech), or anti-*O*-GlcNAc (1:200; Thermo Fisher Scientific, Waltham, USA) primary antibodies. After washing in phosphate-buffer saline (PBS), sections were incubated with peroxidase-labeled secondary antibody for 1 h at room temperature. Sections were then incubated with diaminobenzidine, washed, and counterstained with hematoxylin. All stains were examined by pathologists and semi-quantitatively scored as follows: 0 (no staining), 1 (weakly positive), 2 (moderately positive), and 3 (strongly positive); percentage scores were 0 (0%), 1 (≤10%), 2 (11–50%), and 3 (51–100%). The IHC score for each specimen represents the intensity score multiplied by the percentage score, which ranged from 0–9.

### Statistical analysis

We used time-dependent ROC curve analysis (performed using R, v.3.4.3; Institute for Statistics and Mathematics, Vienna, Austria) with the survivalROC package to select the optimal cut-off value on the basis of the area under the ROC curve (AUC) [26]. We used Spearman rank correlation analysis, the independent samples *t*-test, and the chi-squared test (performed using SPSS v.17.0; SPSS Inc., Chicago, IL) to assess associations between OGT, OGA, or *O*-GlcNAc staining and patient clinicopathological parameters. Survival curves were plotted using the Kaplan–Meier method and compared using the log-rank test. We performed univariate and multivariate analyses using the Cox regression model to investigate the value of clinicopathologic factors for predicting death and tumor recurrence. Differences were considered significant at *P*<0.05.

## SUPPLEMENTARY MATERIALS FIGURES AND TABLE


