# Supplementary material for: High O-linked N-acetylglucosamine transferase expression predicts poor survival in patients with early stage lung adenocarcinoma
Source: Oncotarget. 2018 Jul 24;9(57):31032–44. doi: 10.18632/oncotarget.25772 (PMC6089550; doi:10.18632/oncotarget.25772)
Supplement: Supplementary file 1 [file oncotarget-09-31032-s001.pdf]

High *O*-linked *N*-acetylglucosamine transferase expression predicts poor survival in patients with early stage lung adenocarcinoma

SUPPLEMENTARY MATERIALS

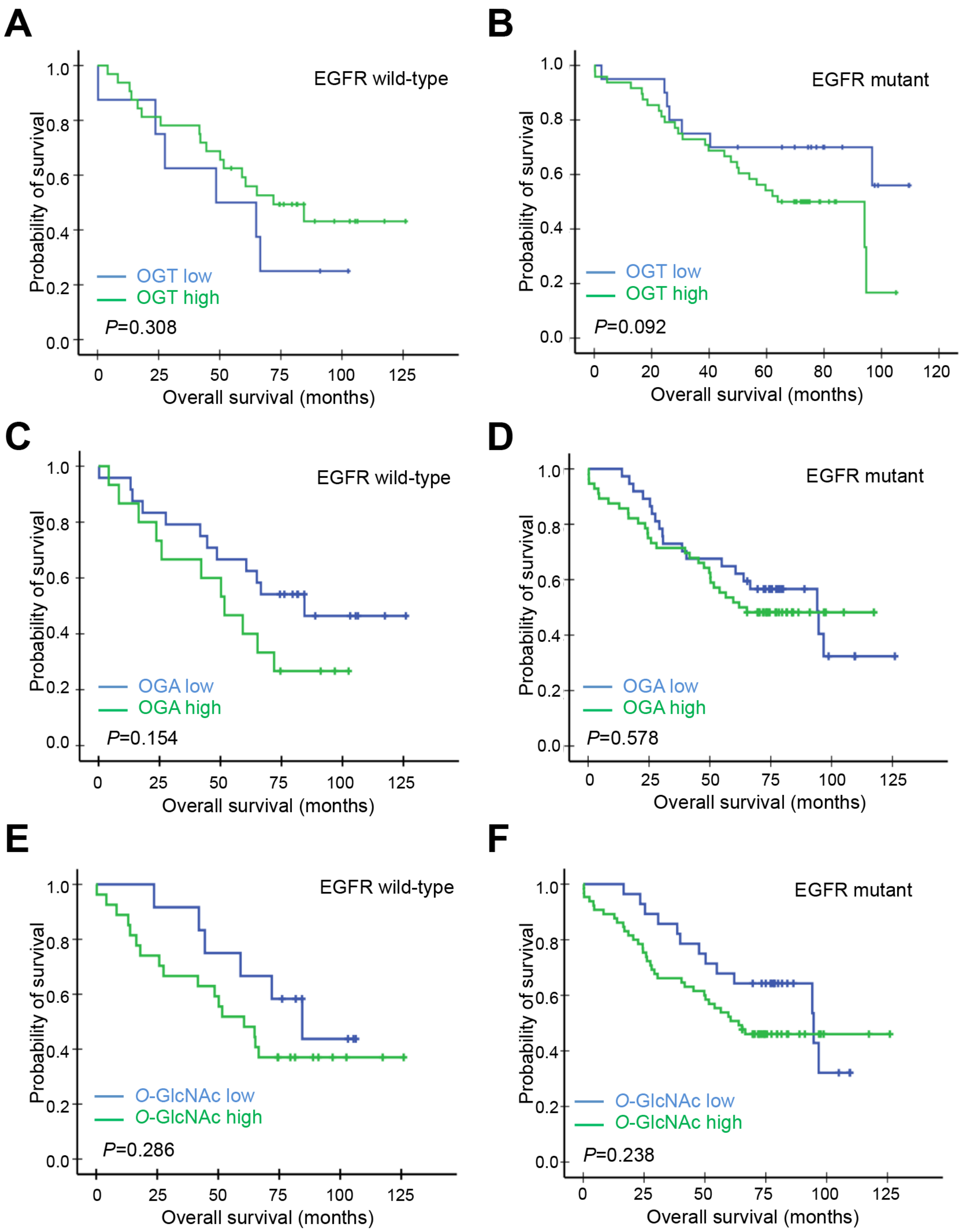

Supplementary Figure 1: Kaplan–Meier survival analysis of patients in cohort A in relation with the *EGFR* status. Patients in cohort A were divided into *EGFR* wild-type **(A, C** and **E)** and *EGFR* mutant **(B, D** and **F)** groups according to the *EGFR* statuses. Each group was further dichotomized by cut-off IHC scores (estimated from time-dependent ROC curves at $t = 120$ months) into high and low OGT (A and B), OGA (C and D) or *O*-GlcNAc subgroups (E and F). OS curves of the groups were plotted; *P* values derived from the log-rank test were indicated in each comparison.

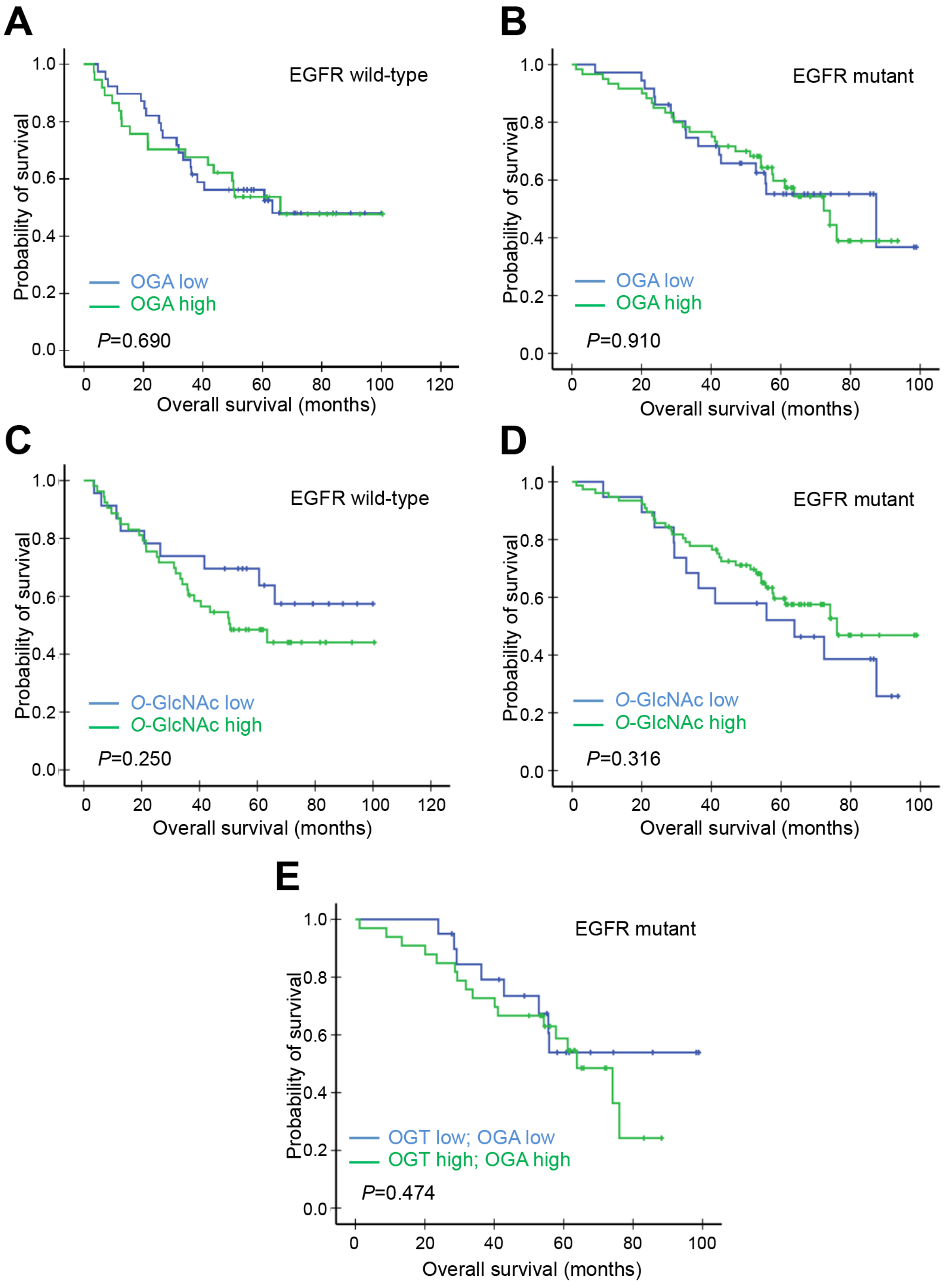

Supplementary Figure 2: Kaplan–Meier survival analysis of patients in cohort B in relation with the *EGFR* status. Patients in cohort B were divided into *EGFR* wild-type **(A** and **C)** and *EGFR* mutant **(B, D** and **E)** groups according to the *EGFR* statuses. Each group was further dichotomized by cut-off IHC scores (estimated from time-dependent ROC curves at $t = 120$ months) into high and low OGA (A and B) or *O*-GlcNAc subgroups (C and D). OS curves of the groups were plotted; *P* values derived from the log-rank test were indicated in each comparison. (E) Patients in the *EGFR* mutant group whose tumors expressed OGT and OGA both at high levels or both at low levels were compared.

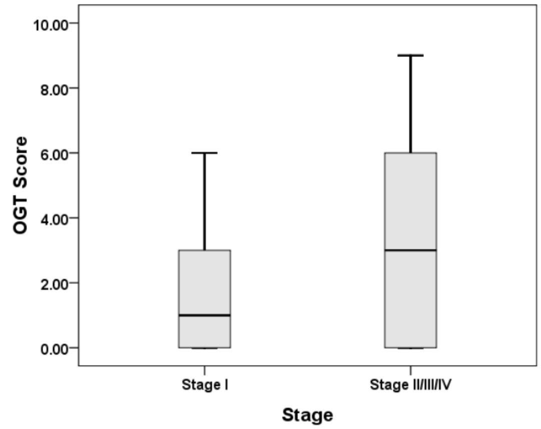

Supplementary Figure 3: OGT expression is higher in stage II/III/IV tumors than in stage I tumors in cohort B.

Supplementary Table 1: Univariate and multivariate Cox regression analysis of recurrence-free survival and overall survival of patients in cohort B

Characteristics	Comparison	Univariate analysis		Multivariate analysis	
		HR (95%CI)	*P* values	HR (95%CI)	*P* values
**Recurrence-Free Survival**					
Age	≦65 years; >65 years	1.204 (0.599-2.423)	0.602		
Gender	Male; Female	0.839 (0.414-1.702)	0.627		
Disease stage group	Stage I; Stage II/III/IV	6.125 (2.936-12.782)	0.000**		
Histological subtype group	Lepidic/acinar/ papillary; Micropapillary/solid	2.124 (0.998-4.520)	0.051		
EGFR status	Wild-type; Mutant	1.061 (0.504-2.232)	0.876		
OGT	Low; High	0.888 (0.443-1.778)	0.737		
OGA	Low; High	1.167 (0.576-2.365)	0.668		
O-GlcNAc	Low; High	1.023 (0.483-2.167)	0.953		
**Overall Survival**					
Age	≦65 years; >65 years	1.272 (0.845-1.915)	0.249		
Gender	Male; Female	0.615 (0.403-0.939)	0.024*	0.522 (0.336-0.811)	0.004**
Disease stage group	Stage I; Stage II/III/IV	3.808 (2.467-5.877)	0.000**	3.821 (2.424-6.021)	0.000**
Histological subtype group	Lepidic/acinar/ papillary; Micropapillary/solid	1.893 (1.242-2.886)	0.003**	1.398 (0.899-2.172)	0.137
EGFR status	Wild-type; Mutant	0.845 (0.546-1.308)	0.450		
OGT	Low; High	1.580 (1.045-2.387)	0.030*	1.332 (0.875-2.030)	0.182
OGA	Low; High	1.605 (1.045-2.466)	0.031*	1.555 (0.998-2.422)	0.051
O-GlcNAc	Low; High	1.040 (0.657-1.648)	0.867		

Abbreviations: HR, hazard ratio; 95% CI, 95% confidence interval. *, $P<0.05$; **, $P<0.01$
